# Pharmacodynamic Interaction of *Quercus infectoria* Galls Extract in Combination with Vancomycin against MRSA Using Microdilution Checkerboard and Time-Kill Assay

**DOI:** 10.1155/2012/493156

**Published:** 2012-07-31

**Authors:** Dayang Fredalina Basri, Radhiah Khairon

**Affiliations:** School of Diagnostic & Applied Health Sciences, Faculty of Health Sciences, Universiti Kebangsaan Malaysia, Jalan Raja Muda Abdul Aziz, 50300 Kuala Lumpur, Malaysia

## Abstract

The galls of *Quercus infectoria* Olivier possess astringent properties which helps in the tightening of the vaginal epithelium in the post-natal period. The present study aimed to observe the time-kill kinetics of the acetone and methanol extracts of gall of *Q. infectoria* in combination with vancomycin against two methicillin-resistant *Staphylococcus aureus* (MRSA) strains; ATCC 33591 and MU 9495 (laboratory-passaged strain). Minimum inhibitory concentration (MIC) of the extracts were determined using microdilution technique whereas the checkerboard and time-kill kinetics were employed to verify the synergistic effects of treatment with vancomycin. The FIC index value of the combinations against both MRSA strains showed that the interaction was synergistic (FIC index <0.5). Time-kill assays showed the bactericidal effect of the combination treatment at 1/8XMIC of the extract and 1/8XMIC of vancomycin, were respectively at 7.2 ± 0.28 hr against ATCC 33591 compared to complete attenuation of the growth of the same strain after 8 hr of treatment with vancomycin alone. In conclusion, the combination extracts of *Q. infectoria* with vancomycin were synergistic according to FIC index values. The time-kill curves showed that the interaction was additive with a more rapid killing rate but, which did not differ significantly with vancomycin.

## 1. Introduction

The prevalence rate and incidence of methicillin-resistant *Staphylococcus aureus* (MRSA) infections are increasing worldwide [1]. Initially, MRSA infections were recorded as the hospital infection MRSA (HA-MRSA) but not long after that, there has been an increase in both community acquired of MRSA infections (CA-MRSA) as well as HA-MRSA [2]. MRSA infection in community settings involves considerable morbidity and mortality, as does nosocomial MRSA infection. In addition, there was a substantial economic burden associated with MRSA in hospitals and these costs will continue to rise if the incidence of MRSA increases further [3]. As such, research to discover an alternative option to cure and prevent MRSA infection is still in full swing. The antibiotic of last resort, vancomycin is only administered when all other treatments fail because there are reports of antibiotic resistance to bacterial strains of MRSA [4]. However, vancomycin is known to be cause nephrotoxicity during its course of administration [5].

 Malaysia is a country rich in biodiversity of plant species thought to have a medicinal potential. Prior to the emergence and development of modern medicine, herbs were considered to be therapeutic agents [6]. *Quercus infectoria* is an oak tree of the family Fagaceae in the Mediterranean area, especially in Greece, Syria, Iran, and Asia Minor [7]. The galls arise on young branches of this tree as result of attack by female gasp-wasp *Adleria gallae-tinctoria* and *Cynips gallae tinctoria* by deposition of the eggs [8]. Traditionally, galls are used in postpartum practice [6] and in the treatment of diarrhea, hemorrhage, and skin disease [8]. The galls of *Q. infectoria* were documented to possess antibacterial [9, 10], anti-MRSA [11], antiviral [12], antifungus [13], larvacidal [14, 15] antioxidant [16], and anti-inflammatory activities [17]. The main constituents of the galls are tannin (50–70%) with small amount of free gallic acid and starch [18]. Previous study showed synergistic interaction between natural products and antibiotics against infectious disease [19, 20]. The present study aimed to assess the synergistic antibacterial activity of the methanol and acetone extracts of *Quercus infectoria* galls in combination with vancomycin against MRSA through time-kill analysis.

## 2. Materials and Methods

### 2.1. Plant Materials

*Q. infectoria* galls were purchased from the local market in Kuala Lumpur and used as plant materials for this study. The specimen was identified in Forest Research Institute Malaysia (FRIM) and deposited there with the voucher no. EZ186/93. The galls were crushed to small pieces using sterile pestle and mortar and powdered in an electric grinder [21].

### 2.2. Preparation of Extracts

The methanol extract was prepared by immersing 100 g of dried material of galls in 500 mL methanol for 24 h at room temperature. The mixture was then filtered and process was repeated using the remaining residue with 300 mL methanol. The two filtrates were combined and concentrated under reduced pressure using a rotary evaporator. The resulting pellet was finally pounded to dryness under hot air-dryer to produce a powdery crude methanol extract [21]. The same procedure was repeated in the preparation of acetone extract.

### 2.3. Preparation of Microorganism

The bacteria used in this study were methicillin-resistant *Staphylococcus aureus* (MRSA) MU  9495, a laboratory-induced strain, and MRSA ATCC 33591 as reference strain. All the bacterial strains were grown and maintained on nutrient agar slants. The inoculum size of each strains was standardized to 10^6^ bacteria/mL for each test by adjusting the optical density of the bacterial suspension to a turbidity corresponding to spectrophotometric absorbance of 0.08 at 620 nm.

### 2.4. Determination of MIC

The minimum inhibitory concentration (MIC) of extracts against MRSA was determined using twofold serial dilution in a 96-well microtiter plate, at final concentration of extracts ranging from 5 mg/mL to 0.0049 mg/mL. The tested extracts were pipetted onto the sterile Mueller-Hinton broth before the diluted bacterial suspension at final inocula of 10^6^ bacterial/mL was added. The bacterial suspension and Mueller-Hinton broth were used as positive control and extracts in Mueller-Hinton broth were used as negative control. The MIC values were taken as the lowest concentration of the extracts in the wells of the microtiter plate that showed no turbidity after 24 hours of incubation at 37°C. The turbidity of the wells in the microtiter plate were interpreted as visible growth of the microorganisms.

### 2.5. Checkerboard Dilution Test

The combined effect of methanol extract and acetone extract with vancomycin was evaluated by checkerboard method to obtain the fractional inhibitory concentration (FIC) index [22]. The checkerboard consisted of column in which each well contains the same amount of antimicrobial agents diluted fourfold along *x*-axis. The rows in which each well contained the same amount of the plant extract and its component were diluted fourfold along the *y*-axis on a 96-well plate. The FIC index was calculated according to the equation: FIC index = FIC_A_ + FIC_B_ = (MIC of drug A in combination/MIC of drug A alone) + (MIC of drug B in combination/MIC of drug B alone). Synergism was defined as an FIC index ≤0.5, the additive effect as an FIC index of 0.5–2.0, and antagonism as an FIC index ≥2.0 [22].

### 2.6. Time-Kill Assay

The time-kill curves of the extracts in combination with vancomycin were evaluated using the microbroth dilution assay. The microtiter wells containing 0.04 mL Mueller-Hinton broth to which the tested combined agents had been added were inoculated with 0.05 mL suspension of the bacterial inoculum. The growth control wells comprised only bacteria and 0.05 mL Mueller-Hinton broth. The wells were then incubated at 37°C and viable counts were performed at 0, 2, 4, 6, 8, and 24 hr after addition of treatment agents. At each hr, 0.01 mL of the sample removed from the wells was diluted twofold with normal saline (0.9% NaCl) and spread on Mueller-Hinton agar plates using L-shaped rod and incubated for 24 hr at 37°C. Colony count of bacteria between 30–300 CFU/mL for each plate was determined to obtain time-mortality curves by plotting the log_10_ CFU/mL on the *x*-axis and time (hr) on the *y*-axis. The interactions were considered synergistic if there was a decrease of ≥2 log_10_ CFU/mL in colony counts after 24 hr by the combination compared to the most active single agent [23]. Additive or indifference was described as a <2 log_10_ CFU/mL, change in the average viable counts after 24 hr for the combination, in comparison to the most active single drug [24]. Antagonism was defined as a ≥2 log_10_ CFU/mL increase in colony counts after 24 hr by the combination compared to that by the most active single agent alone [25]. A combination was considered bactericidal if it produced a 3-log_10_ reduction in colony counts during incubation period denoting >99.9% killing [26].

## 3. Results

### 3.1. Determination of MIC and FIC Index Values

The MIC values of the methanol and acetone extracts from the galls of *Q. infectoria* against MRSA ATCC 33591 and MRSA MU 9495 are shown in Table 1. The MIC values of methanol and acetone extracts against ATCC 33591 were, respectively, 0.625 mg/mL and 0.3125 mg/mL whereas the MIC values of both the extracts were the same (0.3125 mg/mL) against MRSA MU 9495. On the other hand, the MIC values of vancomycin against ATCC 33591 and MU 9495 were much lower, that is, 0.00391 mg/mL and 0.25 mg/mL, respectively (Table 2). The FIC index of the methanol extract and acetone extract in combination with vancomycin against both MRSA strains (0.2498 and 0.1874), which were <0.5 indicating synergistic interaction. All of these synergistic interactions (except for acetone extract against ATCC strain) showed an eightfold decrease in MIC of vancomycin from 0.00391 to 0.000489 mg/mL, whereas acetone extract of *Q. infectoria* galls remarkably reduced MIC of vancomycin by sixteenfold to 0.000244 mg/mL against the reference strains.

### 3.2. Analysis of Time-Kill Kinetics

**Figure 1:**
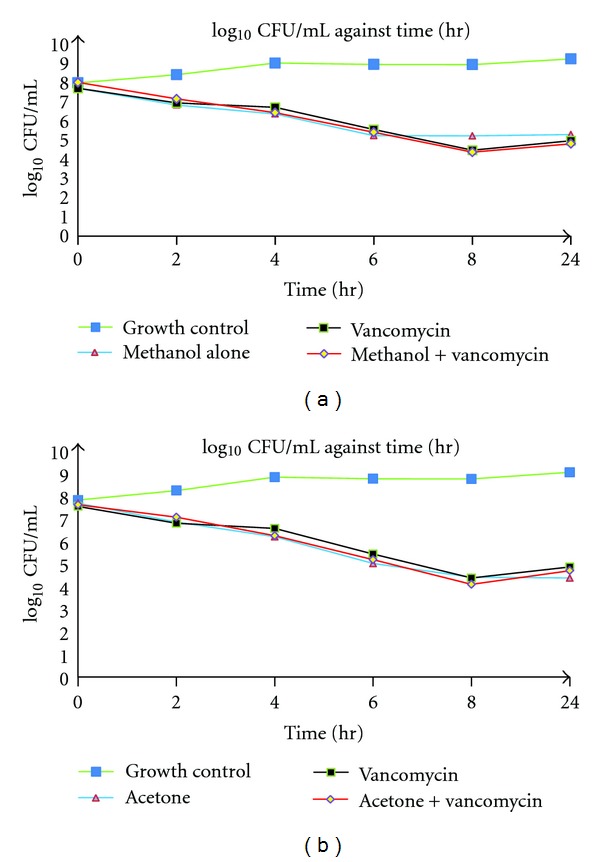
(a) Graph showing time-kill curves of combination of methanol extract with vancomycin, methanol alone, and vancomycin alone against MRSA ATCC 33591. (b) Graph showing time-kill curves of combination of acetone extract with vancomycin acetone alone, and vancomycin alone against MRSA ATCC 33591.

The bactericidal effect of the extracts in combination with vancomycin against MRSA strains was confirmed by time-kill curve experiments. The methanol extract and acetone extracts resulted in a rate of killing at 7 hr and 7.4 hr, respectively, in combination with vancomycin against ATCC 33591 compared with 8 hr by vancomycin alone (Figures 1(a) and 1(b)). This suggests that the combination treatments exerted a stronger bactericidal effect although the difference was not significant.

**Figure 2:**
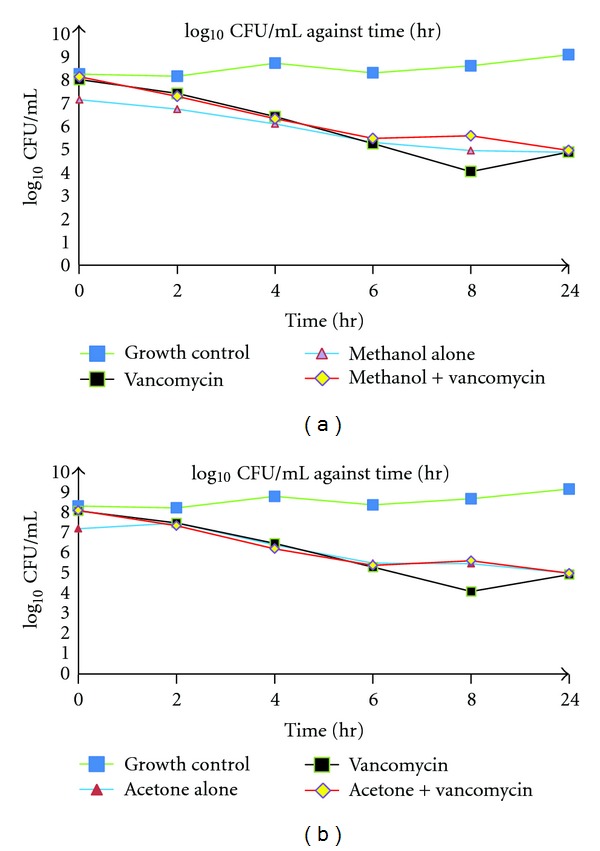
(a) Graph showing time-kill curves of combination of methanol extract with vancomycin, methanol alone, and vancomycin alone against MRSA MU 9495. (b) Graph showing time-kill curves of combination of acetone extract with vancomycin, acetone alone, and vancomycin alone against MRSA MU 9495.

On the other hand, as far as MU 9495 strain was concerned, the synergistic effect of the combination treatment did not display a more rapid killing at 22 hr, compared to vancomycin alone, at 7.2 hr (Figures 2(a) and 2(b)). The time-kill curves also showed that the interaction of all combination of extracts with vancomycin were additive against both MRSA strains.

## 4. Discussion

Nowadays, natural plant and its derivatives are developed as pharmaceutical agents for the treatment of various infections [11, 27]. Synergistic interaction between plant extracts and antibiotics against infectious disease had been reported [19, 20]. Previous research [28] employed well-diffusion method to assess synergism between antimicrobial agents with plant extracts based on enlargement of combined inhibition zone size exceeding 5 mm. However, the checkerboard microdilution technique and time-kill assay were used in the present study to evaluate the antimicrobial effect of antibiotic used in combination with crude plant extracts because they provide detailed information on the type of interaction as well as their bactericidal activity [23]. Checkerboard assay was used to measure the inhibition activity while time-kill study was used to assess bactericidal activity which were dependent on time instead of being concentration dependent [23].

In the present study, the interaction of a combination of methanol and acetone extracts of *Q. infectoria* galls with vancomycin against both MRSA MU 9495 strain and ATCC 33591 strain was synergistic by the FIC index using checkerboard method, but time-kill assay detected additive effect of the combinations against MRSA. This was similar to a report which showed that there was additive activities of acetone extract of *Garcinia kola* seeds with antibiotics [29] and methanolic extract of *Helichrysum pedunculatum* in combination with antibiotics against *Staphylococcus aureus* using time-kill study [30]. The active components of the essential oil of *Thymus vulgaris* also displayed additive antimicrobial activity when time-kill assay was used to verify the observed effects of a “border case of synergism” by FIC test [31].

The discrepancy in the combined evaluation using these two techniques is supported by an observation [32] that summarized most studies which showed contradictory results regarding the comparability of results generated by these two techniques. However, our results were similar to a study that demonstrated that the checkerboard method produced significant synergies for the flavanonol rhamnoside in combination with levofloxacin, but changed to additivity in the time-kill dynamic confirmation test against MRSA [33]. The same finding was observed for the bisbenzylisoquinoline alkaloid in combination with cefazolin against clinical isolates of MRSA [34].

Compared to the two methods of determining the type of interaction between plant extracts and antibiotic, the result obtained by time-kill assay in the present study was favoured over the microdilution checkerboard assay. In fact, the microdilution checkerboard test was not recommended for synergy evaluation [35] and time-kill assays were reported to be more discriminatory than checkerboard titration assays in demonstrating synergy for all combinations [36]. This meant that time-kill methods would be capable of making a finer distinction and produces a more careful judgement compared to the FIC checkerboard methods. Previous research results in our laboratory showed additivity from FIC index values using the checkerboard method against 71.4% of all the total of fourteen MRSA isolates, for both the methanol and acetone combinations with vancomycin [37]. An additive bactericidal effect observed for the combinations in the time-kill experiments could possibly indicate interaction of the crude extracts with common target residues similar to that of vancomycin which lead to competitive inhibition and, consequently, no inhibition of cell growth [38]. The combination of ellagic acid and gallic acid with *β*-lactam antibiotics which resulted in additive mode of interaction against *P. aeruginosa* possibly suggest that these phytochemicals may act at the same target sites in the cytoplasmic membrane with that of ceftazidime and piperacillin [38]. It has been reported that some plant-derived compounds can improve the in vitro activity of some cell-wall inhibiting antibiotics by directly attacking the same target site, that is, peptidoglycan [39]. Polyphenolic compounds have also been shown to exert their antibacterial action through membrane perturbations [30]. High amounts of hydrolysable tannin present in the galls of *Q. infectoria* implied that tannin may be the active compound responsible for the anti-MRSA activity [40]. However, the sustainability test of the methanol and acetone extracts from *Q. infectoria* galls in combination with vancomycin to suppress bacterial growth over a period of time, after a brief exposure of microorganisms to the antimicrobial agents showed a longer postantibiotic effect (PAE) of the combinations compared to that of singly tested extracts and antibiotic [41]. However, this comparison of PAE time only gives an implication for the timing of doses during therapy with antimicrobial combinations against MRSA infections. Evaluation of in vivo effectiveness of the antimicrobial combinations is necessary to generate data that can be extrapolated to the clinical situation as well as to predict relevant concentration of optimal dosing regimens for both agents of the combinations [42].

## 5. Conclusion

It can be concluded that the combination of the extracts from galls of *Q. infectoria* and vancomycin displayed additive bactericidal effect. Furthermore, it also throws light on the possible mechanism of antimicrobial action of the combination treatments which was postulated to be associated with the same target sites of the bacterial cell wall. However, the study of scanning electron microscopic and proteomic analysis of membrane proteins is under way to confirm the nature of the additive combination of anti-MRSA activity of the gall extracts-vancomycin combinations.

## Figures and Tables

**Table 1:** Table showing determination of MIC values of extracts of galls of *Q. infectoria* against MRSA MU 9495 and MRSA ATCC 33591.

Concentration (mg/mL)	MU 9495		ATCC 33591		Control	
	Methanol	Acetone	Methanol	Acetone	Positive	Negative
5.0000	−	−	−	−	+	−
2.5000	−	−	−	−	+	−
1.2500	−	−	−	−	+	−
0.6250	−	−	−	−	+	−
0.3125	−	−	+	−	+	−
0.1563	+	+	+	+	+	−
0.0781	+	+	+	+	+	−
0.0391	+	+	+	+	+	−
0.0195	+	+	+	+	+	−
0.0098	+	+	+	+	+	−
0.0049	+	+	+	+	+	−

**Table 2:** Table showing determination of MIC values of vancomycin against MRSA MU 9495 and MRSA ATCC 33591.

Concentration (mg/mL)	MU 9495	ATCC 33591	Control	
	Vancomycin	Vancomycin	Positive	Negative
1.00000	−	−	+	−
0.50000	−	−	+	−
0.25000	−	−	+	−
0.06250	+	−	+	−
0.03125	+	−	+	−
0.01563	+	−	+	−
0.00781	+	−	+	−
0.00391	+	−	+	−
0.00195	+	+	+	−
0.00097	+	+	+	−
0.00049	+	+	+	−
